# Three functional polymorphisms in *CCDC170* were associated with osteoporosis phenotype

**DOI:** 10.1242/bio.050930

**Published:** 2021-04-16

**Authors:** Xinhong Liu, Yu-Gang Li, Fang Tan, Jia Liu, Ruokun Yi, Xin Zhao

**Affiliations:** 1 Chongqing Collaborative Innovation Center for Functional Food, Chongqing University of Education, Chongqing 400067, China; 2 Chongqing Engineering Research Center of Functional Food, Chongqing University of Education, Chongqing 400067, China; 3 Chongqing Engineering Laboratory for Research and Development of Functional Food, Chongqing University of Education, Chongqing 400067, China; 4 College of Biological and Chemical Engineering, Chongqing University of Education, Chongqing 400067, China; 5 Department of Orthopedics, the First Affiliated Hospital of Chengdu Medical College, Chengdu 610500, China; 6 Department of Public Health, Our Lady of Fatima University, Valenzuela 838, Philippines

**Keywords:** *CCDC170*, miRNA, GWAS lead SNP, Osteoporosis risk

## Abstract

MicroRNAs (miRNAs) play essential roles in regulating bone formation and homeostasis. Genomic variations within miRNA target sites may therefore be important sources of genetic differences in osteoporosis risk. The function of *CCDC170* in bone biology is still unclear. To verify the function of *CCDC170*, we knocked down *CCDC170* in cells and mice and searched for miRNA recognition sites within *CCDC170* using the TargetScan, miRNASNP, and miRBase databases. In this study, our results demonstrated that *CCDC170* plays an important role in the positive regulation of bone formation. MiR-153-3p, miR-374b-3p, miR-4274, miR-572 and miR-2964a-5p inhibited *CCDC170* expression in an allele-specific manner by binding GWAS lead SNPs rs6932603, rs3757322 and rs3734806. These findings may improve our understanding of the association between *CCDC170*, miRNAs, GWAS lead SNPs, and osteoporosis pathogenesis and may provide a potential therapeutic target for osteoporosis therapy.

## Abstract (precis)

**Summary:** CCDC170 may provide a potential therapeutic target for osteoporosis therapy.

## INTRODUCTION

Osteoporosis is a common complex disease defined by bone mineral density (BMD) that is highly heritable, with heritability estimates of 0.5–0.85 (Ralston and Uitterlinden, 2010). With increased age and hormonal changes, the body's bone mass decreases. When bone mass decreases and bone fragility increases, fractures and osteoporosis will eventually occur (Albagha and Ralston, 2003). Fractures due to osteoporosis are a major public health burden (Cauley, 2013). This has led to an increased effort in developing more effective means of treating and preventing bone disease (Sabik and Farber, 2017). Discovering genetically variable loci and clarifying their biological functions in BMD variations are important to understanding the etiology of osteoporosis and developing new approaches to screen, prevent, and treat osteoporosis (Mei et al., 2019).

Single-nucleotide polymorphism (SNP)-based genome-wide association studies (GWASs) have already identified large number of BMD-associated loci (Mo et al., 2015; Cheung et al., 2012; Mullin et al., 2017; Mullin et al., 2016), including *CCDC170*. The CCDC170 protein localizes to the region of the Golgi apparatus and binds Golgi-associated microtubules, and multiple experiments have demonstrated that *CCDC170* plays an essential role in Golgi-associated microtubule organization and stabilization and in the development of breast cancer (Veeraraghavan et al., 2014; Stacey et al., 2010; Villalobos et al., 2017). Multiple GWASs have shown that *CCDC170* is one of the genes most strongly linked to BMD across populations (Hidalgo et al., 2019; Sapkota et al., 2017; Yamamoto et al., 2015). A recent study found that multiple SNPs in *CCDC170* are associated with BMD, including rs6929137, rs371804, rs3734805, rs6932260, rs6904261, rs6932603, rs9383935, rs9383589, rs3734806, and rs3757322 (Qin et al., 2016). The functions in osteogenesis and reconstruction of most of these SNPs and *CCDC170 in vivo* and *in vitro* remain unverified. The present work focused largely on identifying a potential molecular mechanism for *CCDC170*-associated osteoporosis risk and progression.

MicroRNAs (miRNAs) are single-stranded noncoding RNA molecules that are approximately 21–23 nucleotides long and participate in the posttranscriptional regulation of gene expression by directly binding to the 3′ untranslated region (3′-UTR) of target mRNAs (Ambros, 2004; Ameres and Zamore, 2013; Mohr and Mott, 2015; Lou et al., 2018; Paul et al., 2018). MiRNAs play important roles in the occurrence of various diseases including osteoporosis (Poddar et al., 2017; Xie et al., 2017; De et al., 2018; Zhao et al., 2014). In recent years, multiple studies have shown that miRNAs can regulate bone formation and remodeling (Tang et al., 2014; Kim and Lim., 2014; Tu et al., 2017; Cui et al., 2019; Guo et al., 2018).

In order to investigate the role of *CCDC170* and explore the relationship between miRNA, SNPs and *CCDC170* in osteoporosis, we performed a series of gene overexpression and knockdown experiments in cells and mice vivo. In the present study, we found that *CCDC170* could positively regulate bone formation via three GWAS lead SNPs [rs6932603 (C-T), rs3757322 (G/T) and rs3734806 (A/G)]. In addition, we found that miR-153-3p, miR-374b-3p, miR-4274, hsa-miR-572 and hsa-miR-2964a-5p differentially bound to *CCDC170* 3′-UTR to inhibit osteogenesis. In summary, we found a new function of *CCDC170* in bone biology and five new miRNAs that promote the development of osteoporosis, providing new options and targets for the treatment of osteoporosis.

## RESULTS

### Differences in the allele expression of three GWAS lead SNPs

**Fig. 1.**
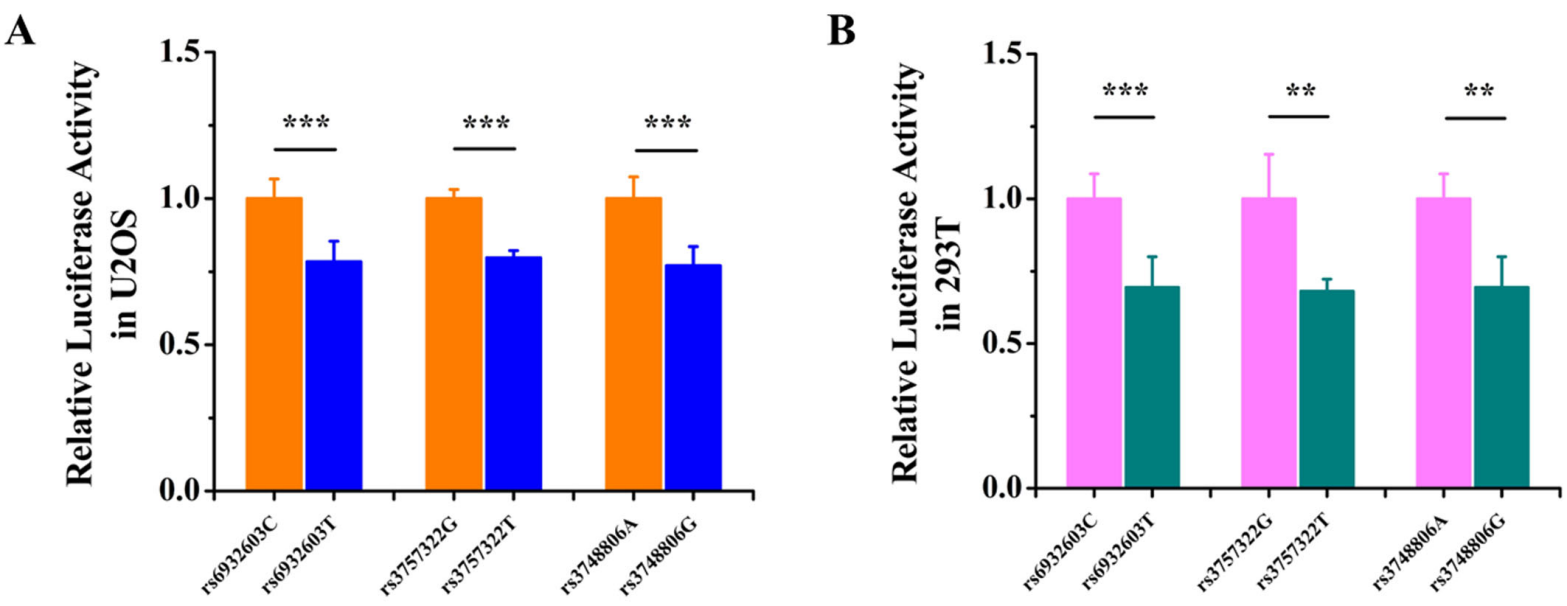
**Differences in the allele expression of three GWAS lead SNPs**. (A) The expression levels of the rs6932603-T, rs3757322-T and rs3748806-G alleles were significantly lower than those of the rs6932603-C, rs3757322-G and rs3748806-A alleles in U2OS cells. (B) The expression levels of the rs6932603-T, rs3757322-T and rs3748806-G alleles were significantly lower than those of the rs6932603-C, rs3757322-G and rs3748806-A alleles in 293T cells. The data are from three independent transfection experiments, with assays performed in triplicate (*n*=6). Firefly luciferase activity was normalized to *Renilla* luciferase activity. The error bars show the standard deviation for six technical replicates of a representative experiment. *P*-values were calculated using a two-tailed Student's *t*-test. **P<0.01, ***P<0.001.

To determine whether the rs6932603-C and rs6932603-T, rs3757322-G and rs3757322-T, rs3734806-A and rs3734806-G allele expression differed, U2OS and 293T cells were transfected with the rs6932603-C, rs6932603-T, rs3757322-G, rs3757322-T, rs3734806-A and rs3734806-G allele psiCHECK-2 vectors. The luciferase reporter assay showed that the rs6932603-T allele expression level was significantly lower than that of the rs6932603-C allele, the rs3757322-T allele expression level was significantly lower than that of the rs3757322-G allele, and the rs3734806-G allele expression level was significantly lower than that of the rs3734806-A allele in the U2OS cells and 293T cells (Fig. 1A,B).

### miRNAs differentially regulated expression of the allele variants of GWAS lead SNPs

**Fig. 2.**
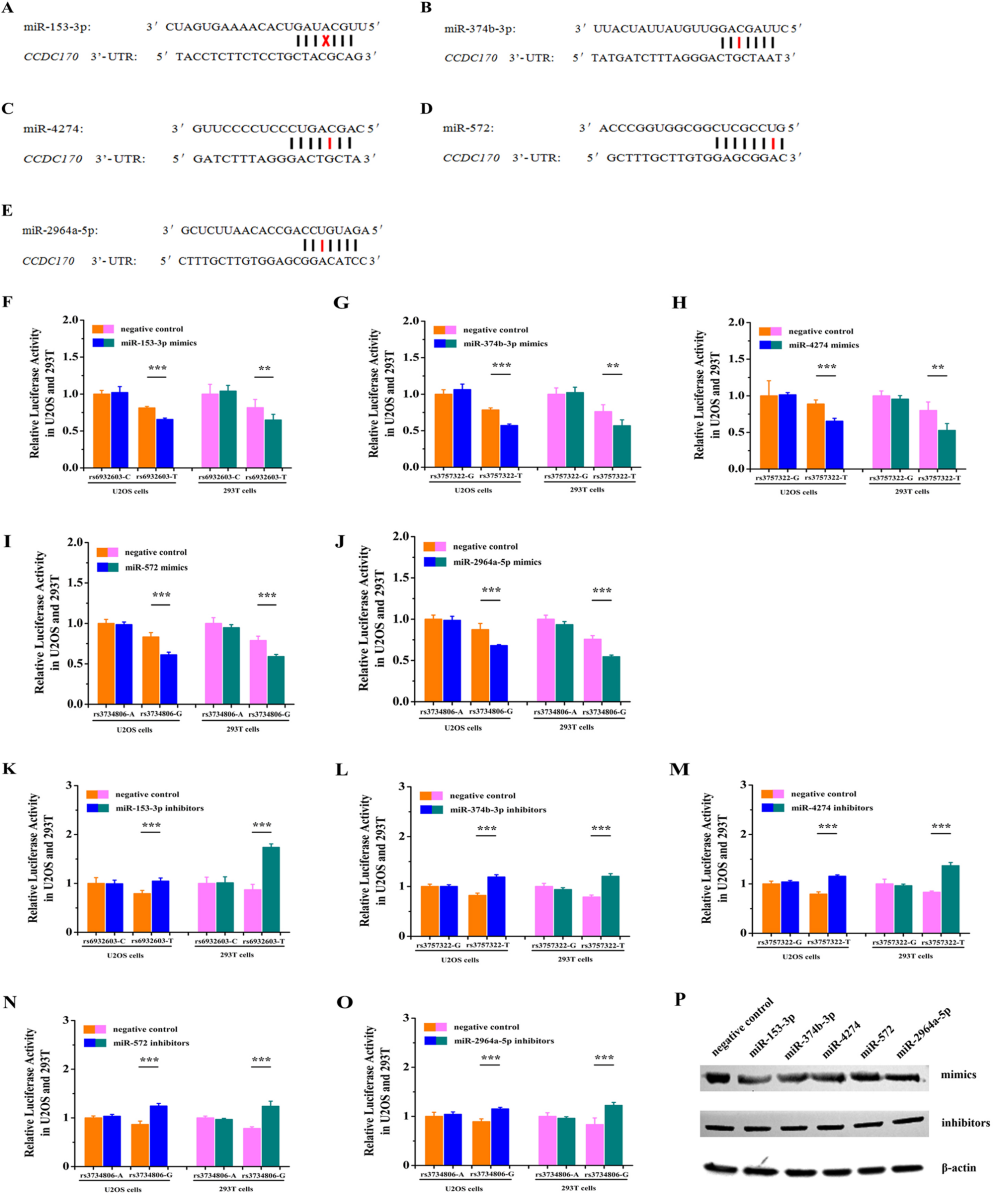
**MiRNAs differentially regulated the allele variants of GWAS lead SNPs.** (A–E) Schematic diagram of the miR-153-3p, miR-374b-3p, miR-4274, miR-572 and miR-2964a-5p binding sites on *CCDC170*. (F) The overexpression of miR-153-3p significantly suppressed rs6932603-T allele levels in U2OS cells and 293T cells. (G,H) The overexpression of miR-374b-3p and miR-4274 significantly suppressed rs3757322-T allele levels in U2OS cells and 293T cells. (I,J) The overexpression of miR-572 and miR-2964a-5p significantly suppressed rs3748806-G allele levels in U2OS cells and 293T cells. (K) Knockdown of miR-153-3p significantly upregulated rs6932603-T allele levels in U2OS cells and 293T cells. (L,M) Knockdown of miR-374b-3p and miR-4274 significantly upregulated rs3757322-T allele levels in U2OS cells and 293T cells. (N,O) Knockdown of miR-572 and miR-2964a-5p significantly upregulated rs3748806-G allele levels in U2OS cells and 293T cells. (P) Overexpression of these five miRNAs can reduce the expression of *CCDC170* at the protein level, while inhibition of this five miRNAs can increase the expression of *CCDC170*. The data are from three independent transfection experiments, with assays performed in triplicate (*n*=6). Firefly luciferase activity was normalized to Renilla luciferase activity. Error bars show the standard deviation for six technical replicates of a representative experiment. *P*-values were calculated using a two-tailed Student's *t*-test. **P<0.01, ***P<0.001.

Next, we tested a biological model in which miR-153-3p differentially regulated the C/T allele variants of rs6932603 in *CCDC170*. In this model, rs6932603-T decreased the *CCDC170* transcription levels, leading to an increased predicted osteoporosis risk (http://mirdsnp.ccr.buffalo.edu/) (Fig. 2A). To test our model, we co-transfected the rs6932603-C/T allele psiCHECK-2 vector and miR-153-3p mimics or NC mimics in U2OS cells and 293T cells. The luciferase reporter assay results showed that the miR-153-3p mimics significantly downregulated rs6932603-T expression in both cell lines (Fig. 2F). As an additional test of our model, the rs6932603-C/T allele psiCHECK-2 vector and miR-153-3p inhibitors or NC inhibitors were co-transfected into 293T cells and U2OS cells. Expression of rs6932603-T was significantly upregulated in both cell lines (Fig. 2K). Using the same prediction method, we found that miR-374b-3p, miR-4274, miR-572 and miR-2964a-5p differentially regulated expression of the allele variants of rs3757322 G/T and rs3734806 A/T in *CCDC170* (Fig. 2B–E). Then, we confirmed that miR-374b-3p mimics and miR-4274 mimics significantly downregulated rs3757322-T expression (Fig. 2G,H), miR-572 mimics and miR-2964a-5p mimics significantly downregulated rs3734806-G (Fig. 2I,J), miR-374b-3p inhibitors and miR-4274 inhibitors significantly upregulated rs3757322-T expression (Fig. 2L,M), and miR-572 inhibitors and miR-2964a-5p inhibitors significantly upregulated rs3734806-G (Fig. 2N,O). Western blot analysis shows that overexpression of these five miRNAs can reduce the expression of *CCDC170* at the protein level, while inhibition of these five miRNAs can increase the expression of *CCDC170* (Fig. 2P). These results indicated that miR-153-3p, miR-374b-3p, miR-4274, miR-572 and miR-2964a-5p inhibited *CCDC170* expression by differentially binding three GWAS lead SNPs.

### miRNA overexpression repressed the expression of osteogenesis marker genes and increased the expression of osteoclastogenesis marker genes

**Fig. 3.**
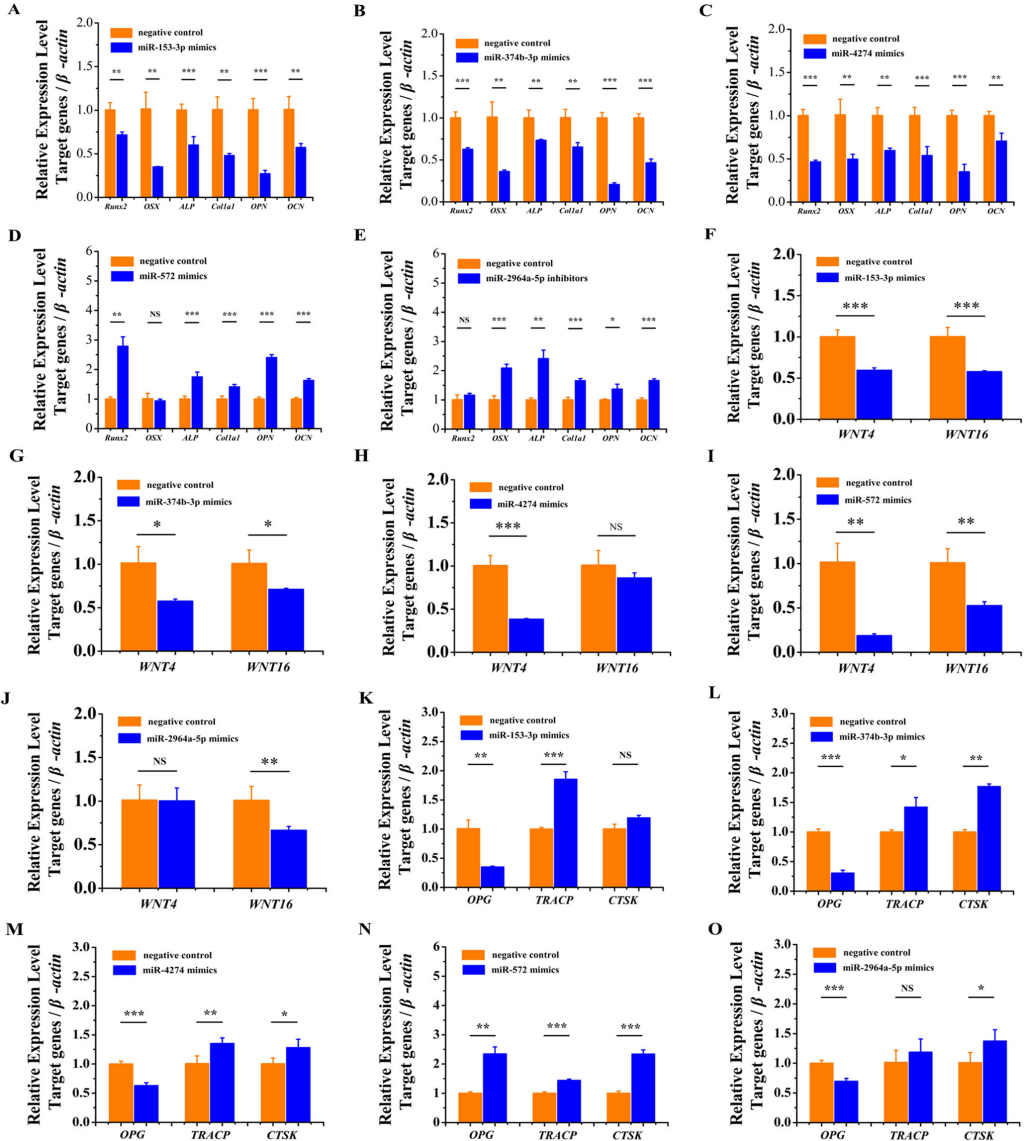
**MiRNA overexpression repressed the expression of osteogenesis marker genes and increased the expression of osteoclastogenesis marker genes.** (A–C) The overexpression of miR-153-3p, miR-374b-3p and miR-4274 significantly suppressed the expression of *Runx2*, *Osterix*, *ALP*, *Col1a1*, *OPN* and *OCN*. (D) The overexpression of miR-572 significantly increased the expression of *Runx2*, *ALP*, *Col1a1*, *OPN* and *OCN*. (E) The overexpression of miR-2964a-5p significantly suppressed the expression of *Osterix*, *ALP*, *Col1a1*, *OPN* and *OCN*. (F,G,I) The overexpression of miR-153-3p, miR-374b-3p and miR-572 significantly suppressed the expression of *WNT4* and *WNT16*. (H) The overexpression of miR-4274 significantly suppressed the expression of *WNT4*, and the expression level of *WNT16* was decreased, but the difference was not significant. (J) The overexpression of miR-2964a-3p significantly suppressed the expression of *WNT16*, but the expression level of *WNT4* did not change. (K) The overexpression of miR-153-3p significantly suppressed the expression of *OPG* and significantly increased the expression of *TRACP*. (L–N) The overexpression of miR-374b-3p, miR-4274 and miR-572 significantly suppressed the expression of *OPG* and significantly increased the expression of *TRACP* and *CTSK*. (O) The overexpression of miR-2964a-5p significantly suppressed the expression of *OPG* and significantly increased the expression of *CTSK*. The expression level of *TRACP* was increased, but the difference was not significant. The data are from three independent transfection experiments, with assays performed in triplicate (*n*=8). The error bars show the standard deviation for four technical replicates of a representative experiment. *P*-values were calculated using a two-tailed Student's *t*-test. NS>0.05, *P<0.05, **P<0.01, ***P<0.001.

To explore the functional role of these five miRNAs in osteosarcoma and osteoporosis, miR-153-3p, miR-374b-3p, miR-4274, miR-572 and miR-2964a-5p mimics were transfected into U2OS cells carrying different alleles of GWAS lead SNPs with NC mimics (Table 1). qRT-PCR results showed that miR-153-3p, miR-374b-3p, miR-4274 and miR-2964a-5p overexpression significantly downregulated the expression of early-stage osteogenesis marker genes *Runx2* and *Osterix*, medium-stage osteogenesis marker genes *ALP* and *Col1a1*, late-stage osteogenesis marker genes *OPN* and *OCN* (Fig. 3A–C,E), and bone formation-related genes *WNT4* and *WNT16* (Fig. 3F–H,J). We also found that when overexpressing miR-153-3p, miR-374b-3p, miR-4274 and miR-2964a-5p osteoclastogenesis inhibitory factor *OPG* was significantly downregulated and that of osteoclastogenesis marker genes *TRACP* and *CTSK* was significantly upregulated (Fig. 3K–M,O). However, miR-572 overexpression significantly upregulated the expression of *Runx2*, *Osterix*, *ALP*, *Col1a1*, *OPN*, *OCN*, *OPG*, *TRACP* and *CTSK* and significantly downregulated the expression of *WNT4* and *WNT16* (Fig. 3D,I,N). ELISA results showed that miR-153-3p, miR-374b-3p, miR-4274 and miR-572 overexpression significantly downregulated the protein content of Runx2 and significantly upregulated the protein content of TRACP (Table 2). These results indicated that miR-153-3p, miR-374b-3p, miR-4274 and miR-2964a-5p overexpression inhibited osteogenesis and promoted osteoclastogenesis.

**Table 1.**
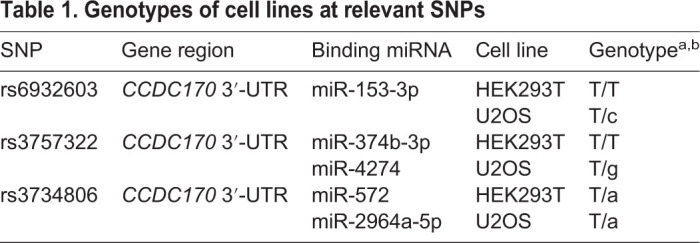
**Genotypes of cell lines at relevant SNPs**

**Table 2.**
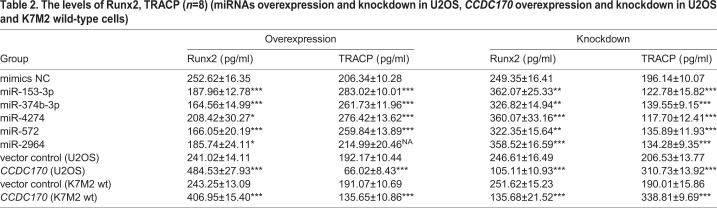
**The levels of Runx2, TRACP (*n*=8) (miRNAs overexpression and knockdown in U2OS, *CCDC170* overexpression and knockdown in U2OS and K7M2 wild-type cells)**

### miRNA knockdown increased the expression of osteogenesis marker genes and repressed the expression of osteoclastogenesis marker genes

**Fig. 4.**
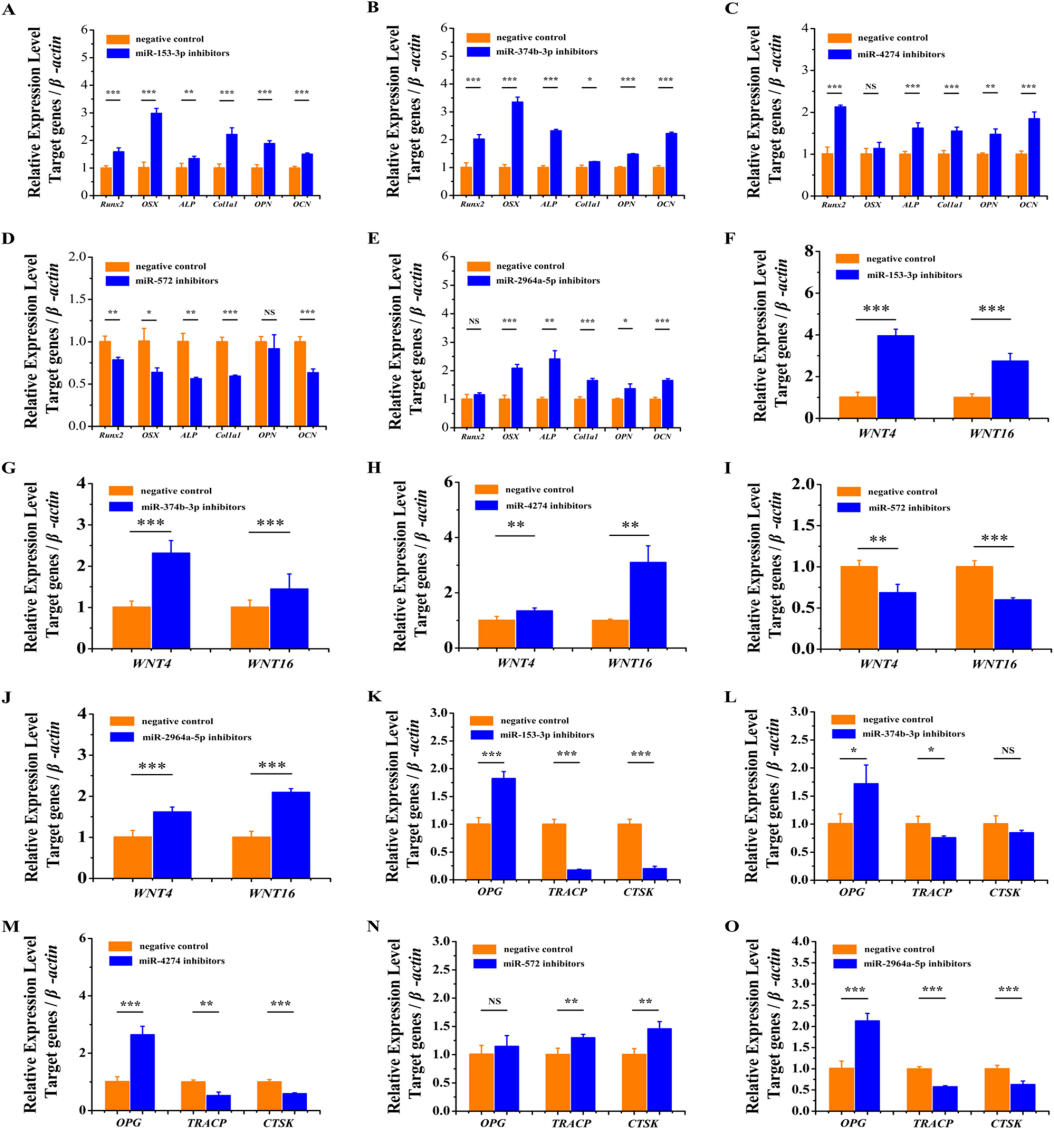
**miRNA knockdown increased the expression of osteogenesis marker genes and repressed the expression of osteoclastogenesis marker genes.** (A,B) Knockdown of miR-153-3p and miR-374b-3p significantly increased the expression of *Runx2*, *Osterix*, *ALP*, *Col1a1*, *OPN* and *OCN*. (C) Knockdown of miR-4274 significantly increased the expression of *Runx2*, *ALP*, *Col1a1*, *OPN* and *OCN*. (D) Knockdown of miR-572 significantly suppressed the expression of *Runx2*, *ALP*, *Col1a1* and *OCN*. (E) Knockdown of miR-2964a-5p significantly increased the expression of *Osterix*, *ALP*, *Col1a1*, *OPN* and *OCN*. (F–H,J) Knockdown of miR-153-3p, miR-374b-3p, miR-4274 and miR-2964a-5p significantly increased the expression of *WNT4* and *WNT16*. (I) Knockdown of miR-572 significantly suppressed the expression of *WNT4* and *WNT16*. (K,M,O) Knockdown of miR-153-3p, miR-4274 and miR-2964a-5p significantly increased the expression of *OPG* and significantly suppressed the expression of *TRACP* and *CTSK*. (L) Knockdown of miR-374b-3p significantly increased the expression of *OPG*, significantly suppressed the expression of *TRACP*, and the expression level of *CTSK* was suppressed, but the difference was not significant. (N) Knockdown of miR-572 significantly increased the expression of *TRACP* and *CTSK*. The error bars show the standard deviation for four technical replicates of a representative experiment. *P*-values were calculated using a two-tailed Student's *t*-test. NS>0.05, *P<0.05, **P<0.01, ***P<0.001.

To further verify the role of these five miRNAs in osteogenesis and osteoclastogenesis, miR-153-3p, miR-374b-3p, miR-4274, miR-572 and miR-2964a-5p inhibitors and non-complementary (NC) inhibitors were transfected into U2OS cells. By qRT-PCR, we found that knockdown of miR-153-3p, miR-374b-3p, miR-4274 and miR-2964a-5p, the expression of *Runx2*, *Osterix*, *ALP*, *Col1a1*, *OPN* and *OCN* was significantly upregulated (Fig. 4A–C,E), that of *WNT4* and *WNT* 16 was significantly upregulated (Fig. 4F–H,J), *OPG* expression was significantly upregulated and *TRACP* and *CTSK* expression was significantly downregulated (Fig. 4K–M,O). When miR-572 was knocked down, the expression of *Runx2*, *Osterix*, *ALP*, *Col1a1*, *OPN* and *OCN* was significantly downregulated (Fig. 4D), that of *WNT4* and *WNT* 16 was significantly downregulated (Fig. 4I), and the expression of *OPG*, *TRACP* and *CTSK* was significantly upregulated (Fig. 4N). ELISA results showed that miR-153-3p, miR-374b-3p, miR-4274, miR-572 and miR-2964a-5p knockdown significantly upregulated the protein content of Runx2 and significantly downregulated the protein content of TRACP (Table 2). These results indicated that knockdown of miR-153-3p, miR-374b-3p, miR-4274 and miR-2964a-5p promoted osteogenesis and inhibited osteoclastogenesis.

### *CCDC170* overexpression increased the expression of osteogenesis marker genes and repressed the expression of osteoclastogenesis marker genes in U2OS and K7M2wt cells

**Fig. 5.**
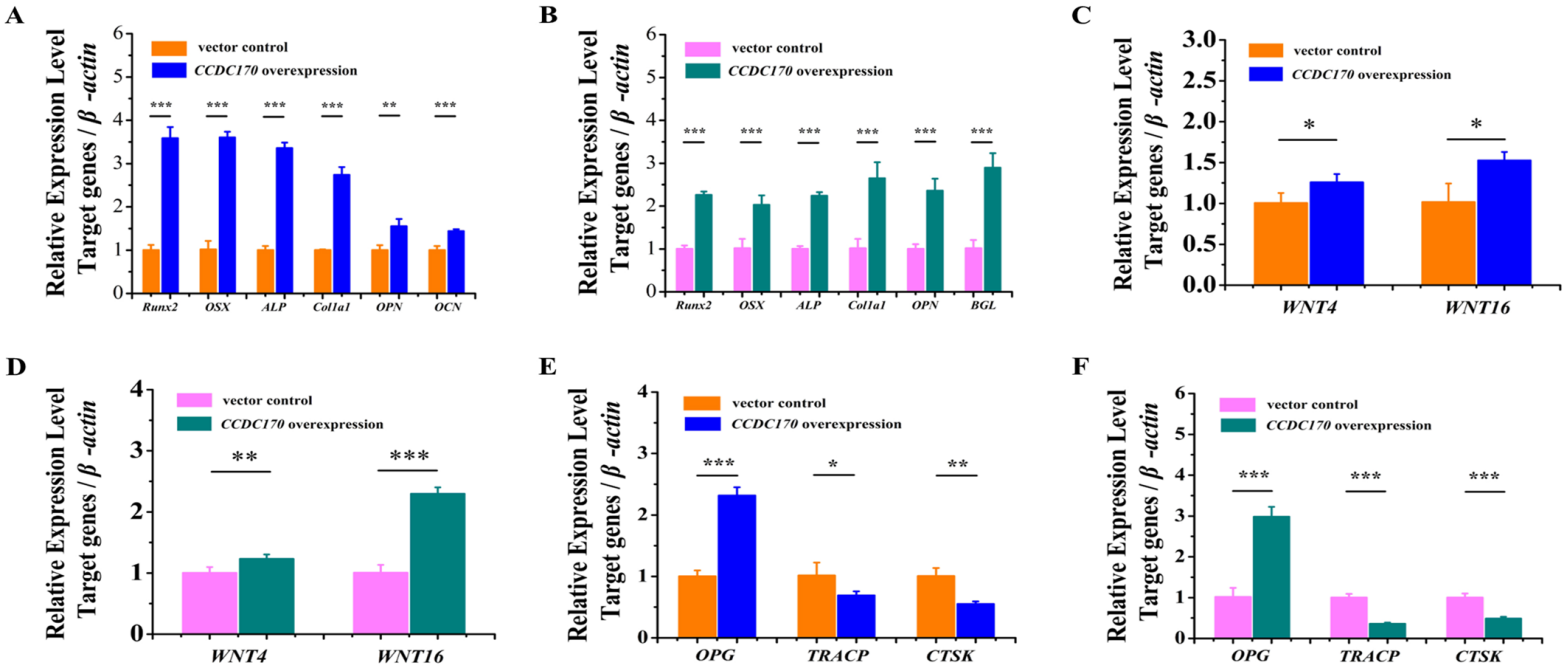
***CCDC170* overexpression increased the expression of osteogenesis marker genes and repressed the expression of osteoclastogenesis marker genes.** (A,B) Overexpression of *CCDC170* significantly increased the expression of *Runx2*, *Osterix*, *ALP*, *Col1a1*, *OPN* and *OCN* (*BGL*) in U2OS cells and K7M2wt cells. (C,D) Overexpression of *CCDC170* significantly increased the expression of *WNT4* and *WNT16* in U2OS cells and K7M2wt cells. (E,F) Overexpression of *CCDC170* significantly increased the expression of *OPG* and suppressed the expression of *TRACP* and *CTSK* in U2OS cells and K7M2wt cells. The data are from three independent transfection experiments, with assays performed in triplicate (*n*=8). The error bars show the standard deviation for four technical replicates of a representative experiment. *P*-values were calculated using a two-tailed Student's *t*-test. * *P*<0.05, ** *P*<0.01, ***P<0.001.

To verify whether the changes in the expression levels of osteogenic and osteoclast markers upon overexpression or inhibition of these five miRNAs are directly caused by changes in *CCDC170*, we overexpressed *CCDC170* in human U2OS cells and mouse K7M2wt cells. By qRT-PCR, we found that the expression of *Runx2*, *Osterix*, *ALP*, *Col1a1*, *OPN* and *OCN* (*BGL*) was significantly upregulated (Fig. 5A,B), the expression of *WNT4* and *WNT16* was significantly upregulated (Fig. 5C,D), the expression of *OPG* was significantly upregulated and the expression of *TRACP* and *CTSK* was significantly downregulated (Fig. 5E,F). ELISA results showed that the protein content of Runx2 was significantly upregulated and the protein content of TRACP was significantly downregulated (Table 2). These results indicated that overexpression of *CCDC170* promoted osteogenesis and inhibited osteoclastogenesis.

### *CCDC170* knockdown repressed the expression of osteogenesis marker genes and increased the expression of osteoclastogenesis marker genes in U2OS and K7M2wt cells

**Fig. 6.**
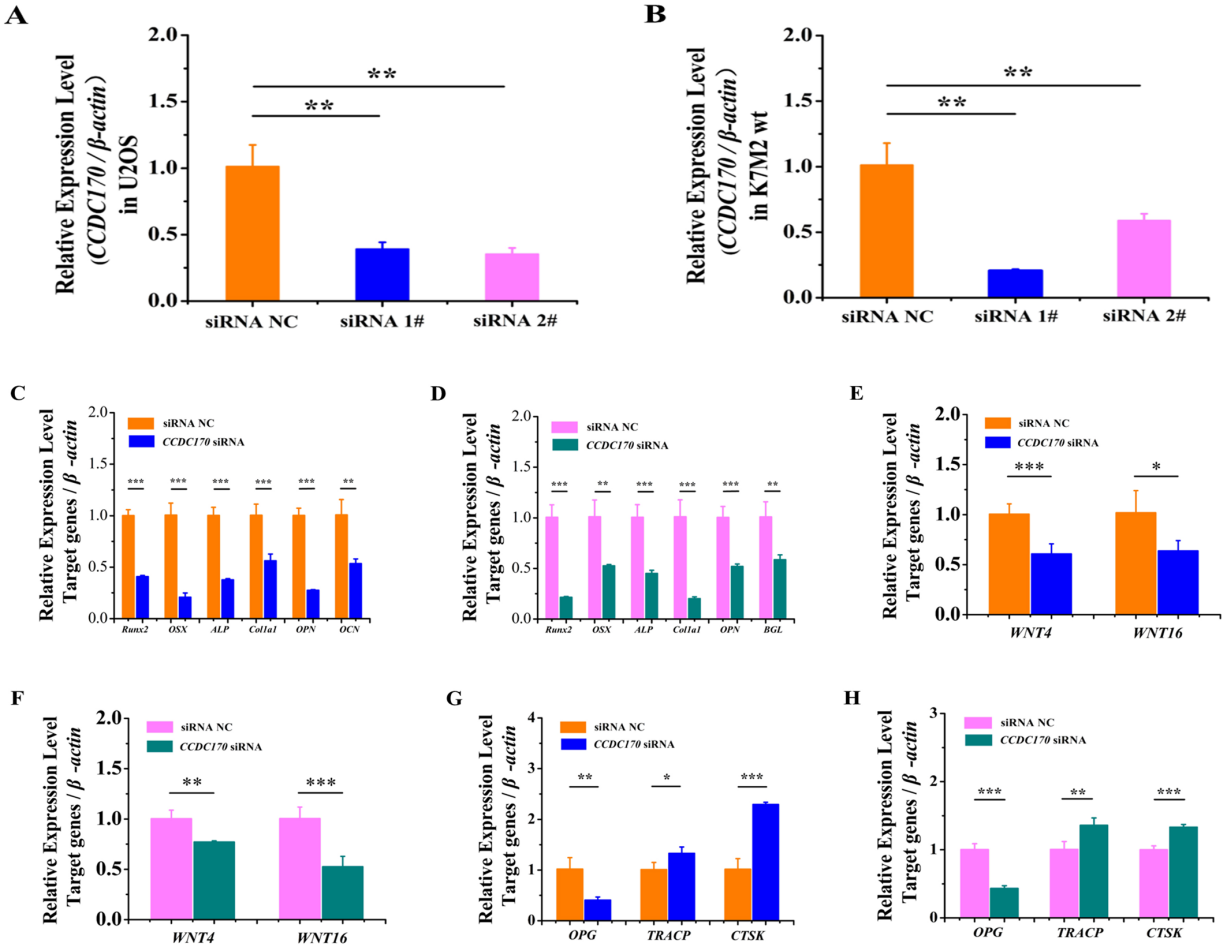
***CCDC170* knockdown repressed the expression of osteogenesis marker genes and increased the expression of osteoclastogenesis marker genes.** (A) Human *CCDC170* siRNA1#, siRNA2# can specifically knockdown the expression of *CCDC170.* (B) Mouse *CCDC170* siRNA1# and siRNA2# can specifically knockdown the expression of *CCDC170.* (C,D) Knockdown of *CCDC170* significantly suppressed the expression of *Runx2*, *Osterix*, *ALP*, *Col1a1*, *OPN* and *OCN* (*BGL*) in U2OS cells and K7M2wt cells. (E,F) Knockdown of *CCDC170* significantly suppressed the expression of WNT4 and *WNT16* in U2OS cells and K7M2wt cells. (G,H) Knockdown of *CCDC170* significantly suppressed the expression of *OPG* and significantly increased the expression of *TRACP* and *CTAK*. The error bars show the standard deviation for four technical replicates of a representative experiment. *P*-values were calculated using a two-tailed Student's *t*-test. *P<0.05, ** *P*<0.01, ***P<0.001.

To further verify the role of *CCDC170* in promoting osteogenesis and inhibiting osteoclastogenesis, *CCDC170* siRNA and siRNA NC were transfected into U2OS cells and K7M2wt cells. By qRT-PCR, we found that human *CCDC170* siRNA1# and siRNA2# and mouse *CCDC170* siRNA1# and siRNA2# could specifically knock down the expression of *CCDC170* (Fig. 6A,B). In follow-up experiments using human *CCDC170* siRNA1# and mouse *CCDC170* siRNA1#, we found that the expression of *Runx2*, *Osterix*, *ALP*, *Col1a1*, *OPN* and *OCN* was significantly downregulated (Fig. 6C,D), the expression of *WNT4* and *WNT16* was significantly downregulated (Fig. 6E,F), the expression of *OPG* was significantly downregulated and the expression of *TRACP* and *CTSK* was significantly upregulated (Fig. 6G,H). ELISA results showed that the protein content of Runx2 was significantly downregulated and the protein content of TRACP was significantly upregulated (Table 2). These results indicated that knockdown of *CCDC170* expression inhibited osteogenesis and promoted osteoclastogenesis.

### *CCDC170* knockdown repressed the expression of osteogenesis marker genes and increased the expression of osteoclastogenesis marker genes in mice vivo

**Fig. 7.**
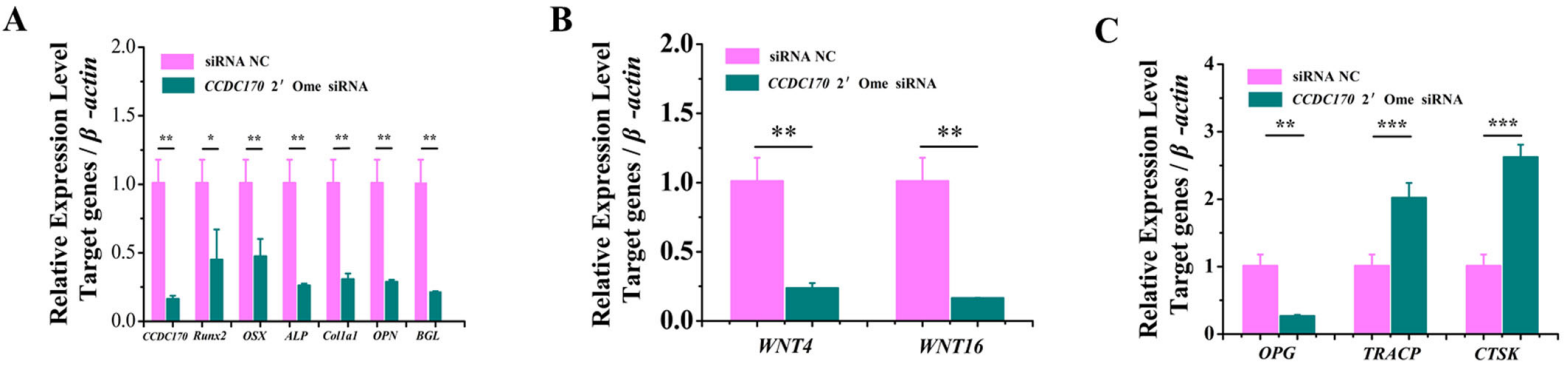
***CCDC170* knockdown repressed the expression of osteogenesis marker genes and increased the expression of osteoclastogenesis marker genes in mice vivo.** (A) Knockdown of *CCDC170* significantly suppressed the expression of *Runx2*, *Osterix*, *ALP*, *Col1a1*, *OPN* and *OCN* (*BGL*) in mice. (B) Knockdown of *CCDC170* significantly suppressed the expression of WNT4 and *WNT16* in mice. (C) Knockdown of *CCDC170* significantly suppressed the expression of *OPG* and significantly increased the expression of *TRACP* and *CTAK*. The error bars show the standard deviation for four technical replicates of a representative experiment. *P*-values were calculated using a two-tailed Student's *t*-test. *P<0.05, ** *P*<0.01, ***P<0.001.

To further verify the role of *CCDC170* in promoting osteogenesis and inhibiting osteoclastogenesis in mice vivo. *CCDC170* 2′-Ome siRNA and siRNA NC were tail vein injected into mice. By qRT-PCR, we found that the expression of *CCDC170*, *Runx2*, *Osterix*, *ALP*, *Col1a1*, *OPN* and *OCN* was significantly downregulated (Fig. 7A), the expression of WNT4 and WNT16 was significantly downregulated (Fig. 7B), the expression of *OPG* was significantly downregulated and the expression of *TRACP* and *CTSK* was significantly upregulated (Fig. 7C). Serum ELISA results showed that the protein content of Runx2, OPG, WNT4 and WNT16 was significantly downregulated and the protein content of TRACP and CTSK was significantly upregulated (Table 2). These results indicated that knockdown of *CCDC170* expression inhibited osteogenesis and promoted osteoclastogenesis in mice vivo.

### *CCDC170* knockdown repressed bone formation in mice

**Fig. 8.**
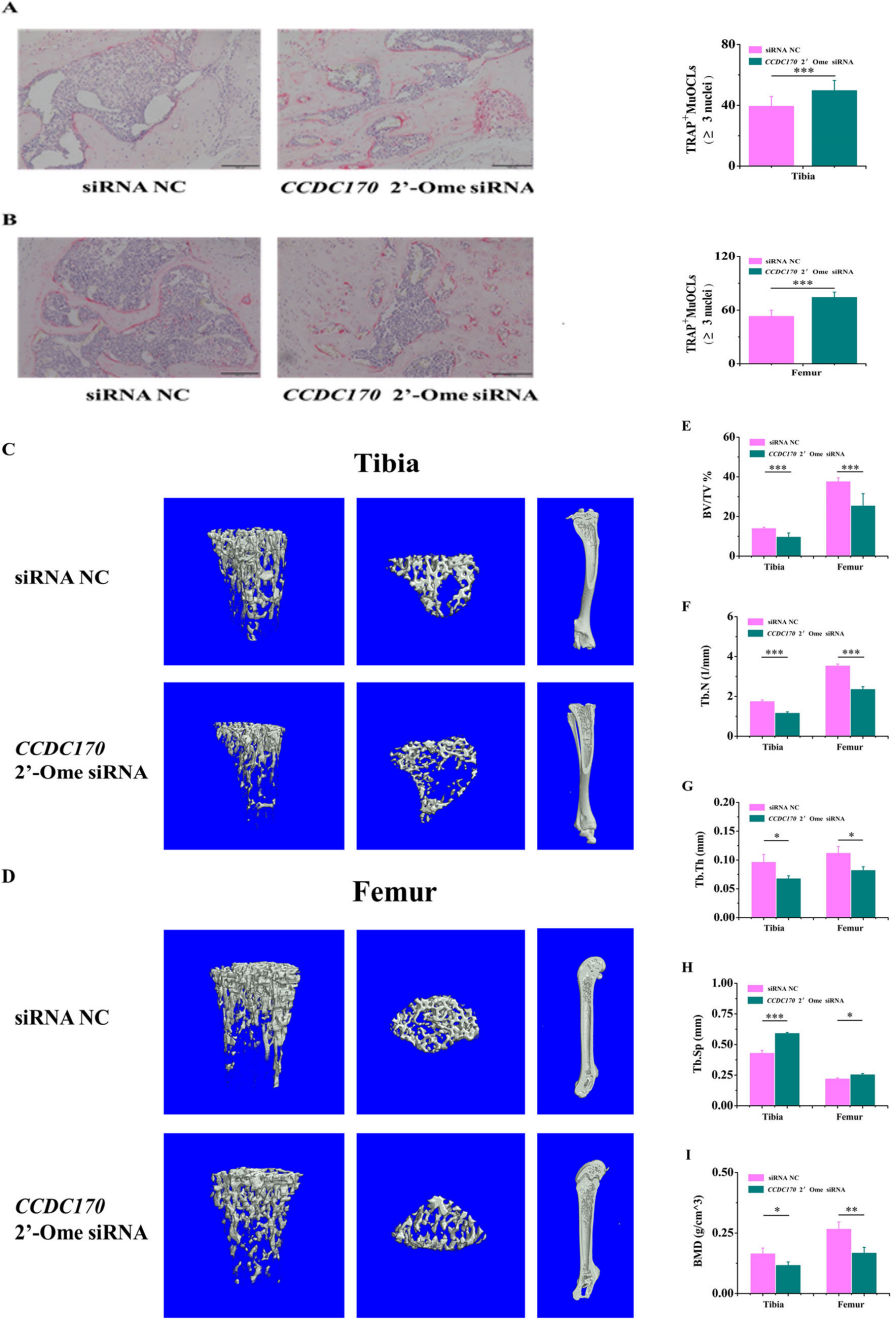
***CCDC170* knockdown repressed bone formation in mice vivo.** (A,B) The number of osteoclasts were increased. (C,D) Representative micro-computed tomography isosurface images. (E) The percent bone volume fraction (BV/TV) was decreased. (F) The trabecular number (Tb.N) was decreased. (G) The trabecular thickness (Tb.Th) was decreased. (H) The trabecular separation (Tb.Sp) was increased. (I) The BMD was decreased. The error bars show the standard of A-B deviation for ten biological replicates of a group and E-I deviation for four biological replicates of a group. *P*-values were calculated using a two-tailed Student's *t*-test. *P<0.05, ** *P*<0.01, ***P<0.001.

To directly evaluate the effect of *CCDC170* in promoting osteogenesis and inhibiting osteoclastogenesis in mice vivo, *CCDC170* 2′-Ome siRNA and siRNA NC were tail vein injected into mice. Ten mice were selected from each group for tibia and femur TRAP staining and osteoclasts count, results showed that *CCDC170* knockdown significantly increased the number of osteoclasts (Fig. 8A,B). Four mice were randomly selected from each group for tibia and femur μCT, all images presented are representative of the respective groups (Fig. 8C,D). The tibia and femur μCT results showed that *CCDC170* knockdown decreased the BV/TV, Tb.N, Tb.Th (Fig. 8E–G) and BMD (Fig. 8I), and increased the Tb.Sp (Fig. 8H).

## DISCUSSION

Identifying target genes is pivotal to understanding the roles of miRNAs in various diseases including osteoporosis (Saetrom et al., 2009; Liu et al., 2015; Juzėnas et al., 2015). The TargetScan, miRNASNP, and miRbase databases were used to identify direct targets of miRNAs. *CCDC170* was predicted to be a target of miR-153-3p, miR-374b-3p, miR-4274, miR-572 and miR-2964a-5p. In our study, we found that these five miRNAs could differentially regulate the allele variants of three GWAS lead SNPs in the *CCDC170* 3′-UTR as demonstrated by the luciferase reporter assay. Our study provides a possible mechanism for this finding, in that upregulation of miR-153-3p, miR-374b-3p, miR-4274, miR-572 and miR-2964a-5p resulted in decreased *CCDC170* expression in osteoporosis.

Multiple studies have found that *CCDC170* plays an important role in breast cancer, and multiple genome-wide linkage analyses have shown that *CCDC170* is one of the genes most strongly linked to BMD (Jiang et al., 2007; Fimereli et al., 2018). However, no *in vivo* or *in vitro* experiments have directly verified the role of *CCDC170*. Our experimental results directly demonstrate that *CCDC170* plays a positive role in bone formation *in vitro* and *in vivo*. *CCDC170* knockdown significantly increased the number of osteoclasts, BMD and other osteogenesis indicators were decreased significantly.

MicroRNAs mediate posttranscriptional regulation of various genes, and some have been shown to influence osteoporosis (Jones et al., 2012; Lian et al., 2015). MiR-153-3p has been shown to play an important role in many diseases, such as lung cancer, breast cancer, and osteosarcoma cell proliferation (Chen et al., 2015; Niu et al., 2015; Zuo et al., 2019). MiR-374b has been shown to play an important role in the proliferation and differentiation of various cell lines (Wu et al., 2018; Jee et al., 2018; Lee et al., 2017; Feng et al., 2011). An association analysis of SNPs located in pri-miRNA sequences with BMD found that miR-4274 overexpression could contribute to the osteoporotic phenotype (De et al., 2017). MiR-572 has been shown to play an important role in renal cell carcinoma, nasopharyngeal carcinoma, and human ovarian carcinoma (Pan et al., 2018; Yan et al., 2017; Zhang et al., 2015). To date, no reports have directly demonstrated the role of miR-153-3p, miR-374b-3p, miR-4274, miR-572 and miR-2964a-5p in the pathogenesis of osteoporosis, while miR-2964a-5p is a new miRNA with no functional reports. Here, we showed that miR-153-3p, miR-374b-3p, miR-4274 and miR-2964a-5p suppressed osteogenesis and promoted osteoclastogenesis by regulating the expression of *CCDC170*. It has been reported that miRNAs have dual functions, and gene expression can be inhibited when the miRNA is located in the cytosol, while gene transcription can be activated when the miRNA is located in the nucleus (Suzuki et al., 2017; Xiao et al., 2017). In our experimental results, miR-572 promoted osteogenesis and suppressed osteoclastogenesis, and we suspect that it activated the expression of another positively regulated bone-forming gene.

## Conclusion

In conclusion, our study demonstrated that *CCDC170* plays an important role in the positive regulation of bone formation and annotated new functions for this gene *in vitro* and *in vivo*. MiR-153-3p, miR-374b-3p, miR-4274, miR-572 and miR-2964a-5p inhibit *CCDC170* expression in an allele-specific manner by binding GWAS lead SNPs rs6932603, rs3757322 and rs3734806. These findings may improve our understanding of the association between *CCDC170*, miRNAs, GWAS lead SNPs, and osteoporosis pathogenesis and may provide a potential therapeutic target for osteoporosis therapy.

## MATERIALS AND METHODS

### Cell cultures

The human embryonic kidney cell line HEK293T was obtained from the Cell Bank of Wuhan University (Wuhan, China). The human osteosarcoma cell line U2OS and the mouse osteosarcoma cell line K7M2wt were purchased from the Cell Bank of the Chinese Academy of Sciences (Shanghai, China). HEK293T cells and K7M2wt cells were cultured in Dulbecco's modified Eagle's medium (DMEM) (HyClone, Waltham, MA, USA), and U2OS cells were grown in McCoy's 5A medium (HyClone). Media were supplemented with 10% fetal bovine serum (Invitrogen, Carlsbad, CA, USA), 100 U/ml penicillin, and 100 μg/ml streptomycin (Invitrogen). All cells were maintained at 37°C in a humidified incubator at 5% CO2.

### Oligonucleotide synthesis and plasmid constructs

MiR-153-3p mimics, miR-153-3p inhibitors, miR-374b-3p mimics, miR-374b-3p inhibitors, miR-4274 mimics, miR-4274 inhibitors, miR-572 mimics, miR-572 inhibitors, miR-2964a-5p mimics, miR-2964a-5p inhibitors, miRNA NC, inhibitor NC, human *CCDC170* siRNA, mouse *CCDC170* siRNA, siRNA NC and SNP genotyping oligonucleotide primers were chemically synthesized (Genepharma, Shanghai, China). The human *CCDC170* and mouse *CCDC170* coding sequences were amplified by whole gene synthesis, and the products were inserted into the pIRES2-EGFP vector (Tsingke, Beijing, China). The inserted sequences were verified by sequencing. Complementary oligonucleotides containing rs6932603-C, rs6932603-T, rs3757322-G, rs3757322-T, rs3734806-A and rs3734806-G were annealed and subsequently cloned into the luciferase reporter vector psiCHECK-2 (Promega, Madison, WI, USA). Vector constructed primers and oligonucleotide sequences are presented in Table S1.

### Genotyping of GWAS SNPs in two cell lines

The PCR-seq method was used to obtain the genotypes of rs6932603, rs3757322 and rs3734806 in U2OS cells and HEK293T cells. A 600-bp sequence centered on rs6932603 (C-T) and a 1000-bp sequence centered on rs3757322 (G/T) and rs3734806 (A/G) were PCR amplified from genomic DNA from the two cell lines using the primers. SNPs rs6932603, rs3757322 and rs3734806 were subsequently genotyped by sequencing the amplified products.

### Cell transfection and luciferase reporter assay

The rs6932603-C, rs6932603-T, rs3757322-G, rs3757322-T, rs3734806-A, and rs3734806-G alleles were amplified and cloned into the psiCHECK-2 vector by inserting them between the *Sgf*I/*Not*I sites. Cells were singly transfected with the SNP allele psiCHECK-2 vector constructs or co-transfected with the SNP allele psiCHECK-2 constructs and miRNA mimics, NC mimics, miRNA inhibitors, or NC inhibitors at 20 nM using Lipofectamine 3000 transfection reagent (Invitrogen) with serum-free media for 48 h per the manufacturer's instructions (the amount of plasmids is 2 µg/well). The luciferase reporter assay was performed using the Dual-Luciferase Reporter Assay (Promega), and the relative luciferase activity was calculated after transfection for 48 h by normalizing the firefly luminescence to that of *Renilla*. Experiments were performed in triplicate, and all experiments were repeated three times.

### RNA extraction and qPCR

Total RNA from spinal cord, U2OS cells and K7M2wt cells was isolated by TRIzol reagent (Invitrogen, Carlsbad, CA, USA). One microliter of oligo(dt)18 primer (500 ng) and 1.0 μl of total RNA (1.0 μg) were added to 10.0 μl of nuclease-free water and heated on a gradient PCR instrument for 5 min at 65°C according to the manufacturer's recommendations (RevertAid First-Strand cDNA Synthesis Kit; Thermo Fisher Scientific, Inc., Waltham, MA, USA). 2 μl of cDNA template was mixed with 10 μl of SYBR Green PCR Master Mix (Thermo Fisher Scientific) and 1 μl upstream and downstream primers. The system was reacted at 95°C for 60 s, then at the conditions of 95°C for 30 s; Annealing Temperature for 30 s; 72°C for 30 s for 40 cycles. Finally, the DNA was detected at 95°C for 30 s and 5°C for 35 s. The 2^−ΔΔCt^ method was used to determine the level of relative gene expression. All primer sequences are presented in Table S2.

### ELISA

The protein content of Runx2, ALP, OPG, RANK and TRACP were determined by using enzyme-linked immunosorbent assay kits (Abcam, Cambridge, MA, USA).

### Western blot analysis

After treating the cells with miRNA mimics and inhibitors for 48 h, the cells were collected. 1×(10^6^) cells were cracked by 1 ml of RIPA and 10 μl of PMSF, then centrifuged at 12,000 r/min at 4°C for 4 min. The intermediate protein layer solution was removed, and the BCA protein quantification kit was used for protein quantification. Samples of each group were diluted to 50 μg/ml, and the diluted protein was mixed with Sample Buffer at the ratio of 4:1 and heated at 100°C for 5 min. Then Mixing Acrylamide, Resolving Buffer, Starcking Buffer, distilled water, 10% APS, and TEMED were mixed in proportion to make SDS-PAGE separation gel and stacking gel, and poured into the gel plate. The Prestained Protein Ladder and the sample were separately added into the sample wells of the gel plate, and the protein-loaded SDS-PAGE gel was subjected to vertical gel electrophoresis for 50 min. The polyvinylidene difluoride (PVDF) membrane was activated by methanol for 1 min and then transmembrane was performed. After that, the PVDF membrane was blocked by 5% fat-free milk containing TBST solution for 1 h. After blocking, the PVDF membrane was washed by TBST. The CCDC170 antibody (Thermo Fisher Scientific, rabbit polyclonal antibody-Catalog Number PA5-55537) was incubated at 25°C for 2 h at a dilution of 1:100, and the second antibody was incubated at 25°C for 1 h. Finally, Supersignal West Pico PLUS was used to fill the PVDF membrane and was placed in the iBright FL1000 (Thermo Fisher Scientific) for observation.

### Animals

Twenty mice from Kunming (six weeks old, female) were purchased from the Experimental Animal Center of Chongqing Medical University (Chongqing, China) The mice were housed in a controlled facility at a constant temperature (25±2°C) and relative humidity (50±5%) with a 12/12 h light/dark cycle and free access to a standard mice chow diet and water. After a 1-week environmental adaptation test, the mice were divided into two groups (negative control group and *CCDC170* siRNA group), ten in each group. Negative control group mice were tail vein injected with 100 μl PBS per week for 4 weeks, *CCDC170* siRNA group mice were tail vein injected the mixture of 100 μl PBS and 50 μg *CCDC170* 2′-OMe siRNA per week for 4 weeks. Three days after the last injection, the mice were fasted for 18 h and then euthanized. Serum was collected from the mice and stored at −80°C for follow-up ELISA experiment. Spinal cord was collected and stored at −80°C for follow-up qPCR experiment. Tibia and femur was collected and fixed in 10% (v/v) buffered formaldehyde for histological observations and microcomputed tomography. The protocol for these experiments was approved by the Ethics Committee of Chongqing Collaborative Innovation Center for Functional Food (201904024B), Chongqing, China.

### Histological observations

The tibia and femur was immediately removed after sacrificing and fixed in 10% (v/v) buffered formaldehyde, paraffin embedded, and sectioned, before tartrate resistant acid phosphatase (TRAP) staining and observation with a BX43 microscope (Olympus, Tokyo, Japan), count the number of osteoclasts with three or more nuclei.

### μCT bone imaging

For micro-computed tomography (μCT) analysis, Bruker MicroCT Skyscan 1272 system (Kontich, Belgium) with an isotropic voxel size of 10.0 μm was used to image the whole tibia and femur. Scans were conducted in 4% paraformaldehyde and used an X-ray tube potential of 60 kV, an X-ray intensity of 166 μA, and an exposure time of 1700 ms. For trabecular bone analysis of the distal tibia and femur, an upper 3 mm region beginning 0.8 mm proximal to the most proximal central epiphysis of the femur was contoured.

### Data analysis and statistics

The data are presented as the mean±s.d. Statistical differences between two groups were determined by two-tailed Student's *t*-test. Statistical differences among groups were analyzed by one-way ANOVA followed by the Student–Newman–Keuls' multiple comparisons test. All experiments were performed independently at least three times with similar results, and representative experiments are shown. *P*<0.05 was considered statistically significant. NS>0.05, *P<0.05, **P<0.01, ***P<0.001.

## Supplementary Material

Supplementary information
