# Supplementary material for: Three functional polymorphisms in CCDC170 were associated with osteoporosis phenotype
Source: Biol Open. 2021 Apr 16;10(4):bio050930. doi: 10.1242/bio.050930 (PMC8061906; doi:10.1242/bio.050930)
Supplement: Supplementary information [file biolopen-10-050930-s1.pdf]

Table S1. Oligonucleotide sequences synthesized in the present study

Name	Sequences (5'–3')
miR-153-3p mimics	UUGCAUAGUCACAAAAGUGAUC
miR-153-3p inhibitors	GAUCACUUUUGUGACUAUGCAA
miR-374b-3p mimics	CUUAGCAGGUUGUAUUAUCAUU
miR-374b-3p inhibitors	AAUGAUAAUACAACCUGCUAAG
miR-4274 mimics	CAGCAGUCCCUCCCCCUG
miR-4274 inhibitors	CAGGGGGAGGGACUGCUG
miR-572 mimics	GUCCGCUCGGCGGUGGCCCA
miR-572 inhibitors	UGGGCCACCGCCGAGCGGAC
miR-2964a-5p mimics	AGAUGUCCAGCCACAAUUCUCG
miR-2964a-5p inhibitors	CGAGAAUUGUGGCUGGACAUCU
NC mimics	UUCUUCGAACGUGUCACGUTT
NC inhibitors	CAGUACUUUUGUGUAGUACAA
rs6932603C-F	CGCAAAAGCCATACCTCTTCTCCTGCTACGCAGAATC TGTTTCTCCTGAATCTC GC
rs6932603C-R	GGCCGCGAGATTCAGGAGAAACAGATTCTGCGTAGC AGGAGAAGAGGTATGGCTTTTGCGAT
rs6932603T-F	CGCAAAAGCCATACCTCTTCTCCTGCTATGCAGAATC TGTTTCTCCTGAATCTCGC
rs6932603T-R	GGCCGCGAGATTCAGGAGAAACAGATTCTGCATAGC AGGAGAAGAGGTATGGCTTTTGCGAT
hsa-*CCDC170* siRNA 1#	GCAGCAACUUUGGUCAAAUTT
hsa-*CCDC170* siRNA 2#	GGAUGCCUCAAAGCAGGAATT
mus-*CCDC170* siRNA 1#	GCACAACACUGAGCUCAAATT
mus-*CCDC170* siRNA 2#	GCUCGAGCACACCAGGAAATT
mus-*CCDC170* 2'-OMe siRNA	GCACAACACUGAGCUCAAATT
siRNA NC	UUCUCCGAACGUGUCACGUTT
rs6932603 genotyping-F	CCATTCACATCAGCATCACT
rs6932603 genotyping-R	GCATCACAGAGATTCAGGAG
rs3757322 genotyping-F	ATTTGACACCTGTTGGTTGG
rs3757322 genotyping-R	GGCATTTTAACAAAGAAGAAAATGG
rs3734806 genotyping-F	ATTTGACACCTGTTGGTTGG
rs3734806 genotyping-R	GGCATTTTAACAAAGAAGAAAATGG

Biology Open • Supplementary information

Table S2. PCR primer sequences in the present study

Name	Sequences (5'–3')	Temperature
hsa-*β-actin*-F	GAGAAAATCTGGCACCACACCT	---
hsa-*β-actin*-R	GCACAGCCTGGATGCAACGTA	---
mus-*β-actin*-F	GGCTGTATTCCCCTCCATCG	---
mus-*β-actin*-R	CCAGTTGGTAACAATGCCATGT	---
hsa-*Runx2*-F	TCTTCACAAATCCTCCCC	52°C
hsa-*Runx2*-R	TGGATTAAAAGGACTTGG	
hsa-*Osterix*-F	CACAGCTCTTCTGACTGTCT	52°C
hsa-*Osterix*-R	GGTGAAATGCCTGCATGGAT	52°C
hsa-*Alp*-F	GACAAGAAGCCCTTCACTGC	59°C
hsa-*Alp*-R	AGACTGCGCCTGGTAGTTGT	59°C
hsa-*Col1a1*-F	CATCTCCCCTTCGTTTTTGA	59°C
hsa-*Col1a1*-R	CCAAATCCGATGTTTCTGCT	59°C
hsa-*OPN*-F	ACTCGAACGACTCTGATGATGT	57°C
hsa-*OPN*-R	GTCAGGTCTGCGAAACTTCTTA	57°C
hsa-*OCN*-F	GCAAGTAGCGCCAATCTAGG	59°C
hsa-*OCN*-R	GCTTCACCCTCGAAATGGTA	59°C
hsa-*OPG*-F	CACAAATTGCAGTGTCTTTGGTC	53°C
hsa-*OPG*-R	TCTGCGTTTACTTTGGTGCCA	53°C
hsa-*TRACP*-F	GACTGTGCAGATCCTGGGTG	63°C
hsa-*TRACP*-R	GGTCAGAGAATACGTCCTAAAAG	63°C
hsa-*CTSK*-F	ACTCAAAGTACCCCTGTCTCAT	62°C
hsa-*CTSK*-R	CCACAGAGCTAAAAGCCCAAC	62°C
hsa-*WNT4*-F	AGGAGGAGACGTGCGAGAAA	60°C
hsa-*WNT4*-R	CGAGTCCATGACTTCCAGGT	60°C
hsa-*WNT16*-F	TTCAGACACGAGAGATGGAACT	63°C
hsa-*WNT16*-R	CCAGCCTTCACTTGCTGAG	63°C
hsa-*CCDC170*-F	TCACGCGGGAGCAGTTAAAC	62°C
hsa-*CCDC170*-R	TCGGAGGTCTTGAAGCTCAGA	62°C
hsa-*Runx2*-F	GTCCCACCATGCACCACCAC	63°C
mus-*Runx2*-R	TTCCGTCAGCGTCAACACCA	63°C
mus-*Osterix*-F	GGAAAGGAGGCACAAAGAAGC	61°C
mus-*Osterix*-R	CCCCTTAGGCACTAGGAGC	61°C
mus-*Col1a1*-F	GCTCCTCTTAGGGGCCACT	63°C
mus-*Col1a1*-R	ATTGGGGACCCTTAGGCCAT	63°C
mus-*OPN*-F	ATCTCACCATTCGGATGAGTCT	60°C
mus-*OPN*-R	TGTAGGGACGATTGGAGTGAAA	60°C
mus-*BGL*-F	AAGCAGGAGGGCAATAAGGTAGT	62°C
mus-*BGL*-R	CCATACTGGTCTGATAGCTCGTCA	62°C
mus-*OPG*-F	CCTTGCCCTGACCACTCTTAT	61°C
mus-*OPG*-R	CACACACTCGGTTGTGGGT	61°C
mus-*TRACP*-F	CACTCCCACCCTGAGATTTGT	63°C
mus-*TRACP*-R	CCCCAGAGACATGATGAAGTCA	63°C
mus-*CTSK*-F	CTCGGCGTTTAATTTGGAGA	60°C
mus-*CTSK*-R	TCGAGAGGGAGGTATTCTGAGT	60°C
mus-*WNT4*-F	AAGAGGAGACGTGCGAGAAAC	60°C
mus-*WNT4*-F	GTCCCTTGTGTCACCACCTT	60°C
mus-*WNT16*-F	CAGGGCAACTGGATGTGGTT	62°C
mus-*WNT16*-F	CTCGTGTCGGAACTGGCTTC	62°C
mus-*CCDC170*-F	ATGCAGAAGGAGTTGAGTGCG	62°C
mus-*CCDC170*-R	AGCTCGTCTCGGGATTGATTT	62°C

Biology Open • Supplementary information
